# Proximate and ultimate causes of pregnancy sickness

**DOI:** 10.1093/emph/eoaf025

**Published:** 2025-09-18

**Authors:** Daniel J Stadtmauer

**Affiliations:** Department of Genetics, Harvard Medical School, Boston, MA, USA; Department of Evolutionary Biology, University of Vienna, Vienna, Wien, Austria

**Keywords:** morning sickness, GDF15, sickness behavior, phylogenetics, placental hormones

## Abstract

Evolutionary biologists have long been fascinated by pregnancy sickness, the heritable, stereotyped syndrome in early pregnancy that usually consists of benign nausea and vomiting and in around 1% of cases progresses to the pathological extreme hyperemesis gravidarum. Identification of the placental hormone GDF15 as a principal causal factor justifies reassessment of its proximate and ultimate causes. This Review synthesizes knowledge of pregnancy sickness at the four levels of analysis of classical ethology—mechanism, development, phylogeny, and adaptive function. Emerging insight into GDF15’s role in innate sickness behaviors suggests pregnancy sickness is a heightened state of pre-existing behavioral defenses triggered by placental production of an emetogenic hormone which may hold a different primary function. Comparison of transcriptomes reveals that placental *GDF15* production rose 100- to 1000-fold to human-like levels in catarrhine primates, and is low or absent in New World monkeys, rodents, and other mammals, with the possible exception of elephants. This suggests that pregnancy sickness is phylogenetically restricted yet not human-specific, and associates with innovations in syncytiotrophoblast biology rather than diet. I re-evaluate leading adaptive hypotheses (prophylactic, metabolic rewiring, placental growth, and anti-rejection) and argue that the key to adjudicating among them hinges on whether GDF15 acts locally through non-canonical receptors and whether additional factors distinguish pregnancy sickness from sickness behavior. Finally, I evaluate explanations for the persistent risk of hyperemesis gravidarum in modern humans, including trade-offs, mismatch, and conflict. With recent advances, pregnancy sickness is not just a curiosity of human evolution, but a compelling opportunity to investigate the mechanistic bases of complex adaptive behaviors.

## INTRODUCTION

Pregnancy sickness refers to the constellation of symptoms experienced during early gestation, most prominently nausea and vomiting in pregnancy (NVP), which frequently co-occur with symptoms like food aversions, cravings, heightened olfaction, and social withdrawal. While frequently used interchangeably, here ‘pregnancy sickness’ will be used to refer to the wider phenomenon, and NVP to refer to just nausea and vomiting. Often dismissed as benign, pregnancy sickness can cause considerable suffering, particularly at the severe end of the spectrum if it escalates to hyperemesis gravidarium (HG), a condition involving fluid and electrolyte imbalance, nutritional deficiency, and weight loss [1]. NVP carries an economic burden exceeding $1.7 billion in hospital admissions in the United States alone [2], and presents a substantial lifestyle burden, being a leading reason for to maternal sick leave [3]. Experience of severe nausea and vomiting is a significant contributor to women’s decisions to terminate pregnancy, and to not pursue subsequent pregnancy [4–6].

NVP is heritable, variable, and common. Most research on NVP comes from industrialized Western populations. A meta-analysis of 79 studies and 93 753 individuals across North America, Europe, Asia, and 2 in Africa estimated that NVP occurs in 69% of pregnancies, with individual study rates ranging from 35% to 91% [7]. This variation likely reflects differences in environment and genetic background as well as methodological factors such as how NVP is defined [8, 9]. Approximately 1.1% of these pregnancies (95% CI 0.8–1.3) progressed to hyperemesis gravidarum [7]. Ethnographic reports from more than 20 non-industrialized, hunter-gatherer and subsistence farming societies confirm NVP’s broad reach, while it is reportedly absent in eight such societies [10, 11]. While more data from undersampled regions is needed to understand its global prevalence, available evidence suggests that the capacity for NVP is a human universal [12].

The peculiar trait of pregnancy sickness has long attracted evolutionary theories. These theories include that it is an adaptation to avoid teratogenic foods [10, 13, 14], that it is a side-effect of maternal mechanisms for embryo quality control [15, 16], and that it is a byproduct of fetal signaling to increase maternal investment [17–19]. Explaining pregnancy sickness requires integrating these ultimate ‘why’ questions with proximate ‘how’ questions [20]. Medical research into NVP has focused on the latter, and evolutionary medicine provides a framework for integrating mechanistic insights into evolutionary models for a more comprehensive understanding of disease [21].

Research into the mechanisms of nausea and appetite loss (anorexia) has revealed that placental production of the peptide hormone growth/differentiation factor 15 (GDF15) is a key driver of NVP [22, 23]. At the same time, the idea that animals have evolved behavioral defenses against toxins and pathogen vectors in non-reproductive contexts has advanced in recent years from the speculative margins of human evolutionary psychology to field ecology [24] and neurobiology [25, 26].

This Review synthesizes recent findings to reassess the origins of pregnancy sickness in light of its newly uncovered neuroendocrine basis. It is organized according to the four levels of analysis of classical ethology [27]: mechanism, development, adaptive function, and phylogenetic origin.

Evolutionary perspectives on pregnancy sickness have implications for intervention. Prophylactic evolutionary narratives are utilized in nutritional guidance and contribute to reluctance to treat NVP [28, 29], and more recently drug development efforts targeting GDF15 often frame NVP as vestigial or primitive to justify intervention [30, 31]. However, GDF15 is not just a nausea hormone. It is a multifunctional signal involved in metabolism, stress responses, immunoregulation, and cellular growth. If placental GDF15’s primary function lies in placental growth or immune tolerance rather than solely appetite modulation, targeting the ligand rather than just the nausea circuit specifically could disrupt primate-specific functions in placental development which would be absent in model organisms. A more complete understanding of both proximate and ultimate causes will support the design of safe and effective interventions.

## MECHANISM

### Nausea and vomiting of pregnancy is proximately triggered by placental GDF15

Pregnancy-associated nausea is triggered by activation of the area postrema, a chemosensitive organ in the brainstem located beyond the blood–brain barrier [32]. Unlike motion sickness, which is initiated by the vestibular system, the area postrema regulates food- and circulating toxin-borne nausea [33]. The area postrema is highly vascularized, with fenestrated capillaries to monitor circulating compounds, integrate peripheral hormone signals, and elicit appropriate neurological responses [34, 35]. Neurons in the area postrema express receptors for peripherally-produced appetite regulators like glucagon-like peptide 1 and amylin as well as GDF15 [36]. GDF15 activates these neurons by binding a cell-surface receptor complex formed by the protein GFRAL and its co-receptor RET [37], whose co-expression is highly restricted to neurons in the area postrema and the adjacent nucleus tractus solitarius [38]. Activation of these neurons induces defensive behaviors including emesis, adipsia, anorexia, reduced novelty-seeking, and reduced ambulatory activity [39, 40].

GDF15 is a divergent member of the transforming growth factor-beta (TGF-β) superfamily, retaining low sequence similarity to canonical TGF-β ligands like TGFB1 and TGFB2. It is produced in diverse physiological contexts, including by the liver after strenuous physical exertion [41, 42], by intestinal enteroendocrine and goblet cells upon ingestion of bitter substances [43], and by activated immune cells during infection [44]. GDF15 plays a role in the cellular integrated stress response [45] and has been implicated in anorexia, cancer cachexia, and chronic inflammatory disease. Unlike homeostatic appetite regulators such as leptin and ghrelin, GDF15 levels do not fluctuate with food intake [46]. It does not directly contribute to bacterial or viral pathogen clearance, either [47]. Instead, GDF15 appears to act allostatically, shifting physiological set points to promote the tolerance of stress and other challenges through indirect and anticipatory defenses [44, 48]. These multifaceted and indirect effects have earned GDF15 the moniker of ‘mystery hormone’ [49].

A direct connection between GDF15 and nausea and vomiting of pregnancy was first identified in a 2018 genome-wide association study based on 23andMe data, which found that a common single-nucleotide polymorphism (rs16982345) associated with reduced maternal *GDF15* expression confers up to an 8-fold increased risk of hyperemesis gravidarum, alongside risk alleles including one affecting GDF15’s receptor *GFRAL* [23]. *GDF15* and *GFRAL* have recurred in subsequent genome-wide association studies [50, 51]. The human placenta produces high levels of GDF15, with levels rising during the first trimester; almost all GDF15 in the maternal circulation during pregnancy is of placental origin, and its levels correlate with the presence of NVP symptoms [22]. These studies provide strong evidence that that placental GDF15 acts through an area postrema-specific circuit to induce nausea.

Further evidence for a causal link of GDF15 to NVP came from Mendelian randomization analyses showing that genetic variants associated with lower pre-pregnancy GDF15 levels significantly increase hyperemesis gravidarum risk [22]. This suggests that maternal baseline levels before pregnancy set the threshold for sensitivity. Indeed, some of the same *GDF15-*enhancing variants have opposing effects on NVP depending on whether they are present in the mother or the fetus: fetal *GDF15* determines circulating levels in pregnancy, whereas maternal *GDF15* determines pre-pregnancy background [50]. Mouse models have shown that prior exposure to GDF15 blunts sickness behaviors during subsequent exposure [22]. Women with lower serum GDF15 levels tend not to vomit during pregnancy [22, 52], yet even in women who do, nausea symptoms typically abate after the first trimester even though GDF15 continues to rise [22, 57–59]. These patterns are consistent with fold-change detection [60], where an output responds to relative changes rather than absolute concentrations of a trigger. Desensitization of the GFRAL/RET receptor in area postrema neurons would explain this pattern [61].

### Syncytiotrophoblast is central to pregnancy-specific endocrine rewiring

**Figure 1:**
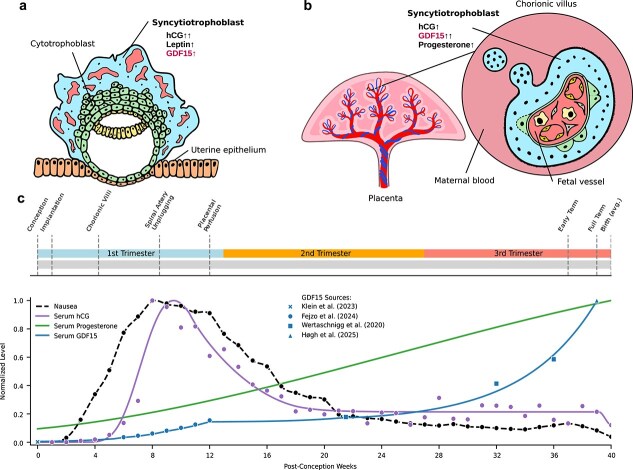
Syncytiotrophoblast is the main placental source of signals affecting maternal neuroendocrine state. (a) Early first-trimester syncytiotrophoblast produces high levels of hCG, leptin, and GDF15. (b) Syncytiotrophoblast in the established placenta surrounds fetal microvasculature in pools of maternal blood, and produces high levels of GDF15 and progesterone, while hCG and leptin production are reduced. (c) Timeline of key events in human pregnancy and trends in circulating hormone levels. Nausea level data points are digitized from [53] (n = 500, London); serum hCG level data points are digitized from [54] (n = 443, USA); serum progesterone levels are plotted as a general trend after [55] (n = 183, Singapore); serum GDF15 levels are plotted from data compiled from multiple sources as noted. Drawings in (**a**) and (**b**) are adapted from [56] under a CC BY 4.0 license.

Human blastocysts begin to express *GDF15* early in development [62], and as the placenta develops, the fusing fetal cells known as syncytiotrophoblast emerge as a cellular source of GDF15. GDF15, the peptide subunits of human chorionic gonadotropin (hCG), leptin, and kisspeptin constitute the most abundantly produced proteins in syncytiotrophoblast [63]. Between weeks 4 and 5, chorionic villi form (Fig. 1a, b) as the syncytiotrophoblast expands and branches into the maternal endometrium [64]. Trophoblast plugs in maternal spiral arteries are dislodged between weeks 8 and 10, allowing greater transfer of placental products into maternal circulation, and by week 12 the placenta is fully perfused with blood [64, 65]. By the end of the first trimester, hCG and leptin levels fall, while both progesterone and GDF15 to continue to climb (Fig. 1c) [66–68]. Extravillous trophoblast, the invasive cells of the placenta that migrate interstitially into maternal tissues, also produce GDF15 to a lesser extent at this time [50, 69].

The mass of placental trophoblast determines GDF15 dosage and NVP severity. Women with trophoblast tumors such as hydatidiform moles [70], and those gestating twins [71], are more likely to have hyperemesis gravidarum. For decades, pregnancy sickness was attributed to hCG due to its temporal correlation with symptom severity (Fig. 1c) [72] and observations of elevated hCG levels in patients with hyperemesis gravidarum [73]. However, because hCG is produced by the syncytiotrophoblast at the same time as GDF15, its relationship to nausea is likely correlation with placental mass, not causation. Another candidate, thyroid hormone (T_4_) [73], is driven by the fact that hCG can activate the thyroid-stimulating hormone receptor to induce thyroid hormone production, rather than directly causal [74].

### Central and peripheral mechanisms of action

Emetogenic effects of GDF15 are mediated by its receptor GFRAL in the brainstem, which remains the best and only rigorously identified biochemical pathway. However, several lines of evidence suggest that GDF15 may also exert direct effects on peripheral tissues, including kidney, liver, and tumors [75, 76]. The lack of a verified receptor by which these effects can be mediated has made GFRAL-independent actions of GDF15 controversial. A comprehensive ligand-binding panel of TGF-β receptor gene family members showed that the TGF-β receptors TGFBR1 and TGFBR2 cannot bind GDF15 directly [77]. Expression of GFRAL in peripheral tissues is unlikely, as extensive searches have found no GFRAL-expressing neurons outside of the area postrema and nucleus of the solitary tract [38]. Thus, peripheral action would require non-GFRAL and non-TGFB1/2 receptors.

One of the non-GFRAL receptors proposed for GDF15 in the immune system is CD48, a cell surface protein on lymphocytes shown to interact with GDF15, but this interaction has not been biochemically characterized [78]. It is also possible that peripheral GDF15 binding is not one-on-one but combinatorial, achieved by a yet-uncharacterized protein complex which activates similar downstream messengers as TGF-β receptors. Evidence for such a multimeric complex comes from evidence that ERBB2 (HER2), a co-receptor from the epidermal growth factor receptor family, mediates GDF15-induced growth signaling in ovarian cancer cells despite lacking its own ligand-binding domain [79]. The limited evidence for non-GFRAL-dependent pathways remain the main barrier to functional explanations of pregnancy sickness that depend upon local effects on uteroplacental biology, as will be discussed below.

Early experiments testing the effects of GDF15 used commercially-available recombinant GDF15 synthesized in mammalian cells, which were later found to contain biologically active levels of TGF-β [80]. As such, proposed GFRAL-independent signaling mechanisms that derive their support from mammalian-sourced recombinant GDF15 require scrutiny to eliminate the possibility that they are TGF-β artifacts.

Despite considerable progress in understanding the molecular mechanisms of pregnancy sickness, critical gaps remain. Does observed variability in NVP severity and diversity of behavioral and physiological changes associated with pregnancy sickness derive solely from variation in GDF15 responses, or are other factors involved? Are nausea and food aversions part of a coordinated adaptive syndrome promoting reproductive success, or an incidental and potentially costly byproduct of the placenta’s production of an emetogenic hormone whose primary function lies elsewhere?

## DEVELOPMENTAL ORIGIN

The developmental level of analysis refers to how a trait is acquired and changes over an individual’s life. This includes the unfolding of genetically determined processes driving innate patterns of behavior and the shaping effects of environment and learning [81]. Developmental analysis of pregnancy sickness must address how the state is triggered hormonally, how that stimulus interacts with maternal physiological state (such as prior pregnancies, metabolic balance, infection and immune status), and how it is formed by learning (e.g. conditioned taste aversion).

NVP has historically been dismissed as a biologically meaningful trait with an ontogenetic trajectory. Because symptoms are variable, largely subjective, and often lack overt pathology, pregnancy sickness has often been cast as psychosomatic or even fabricated, motivated by secondary gain [82]. As recently as the 1990s, psychoanalytic theories of pregnancy sickness were commonplace, which allege that it reflects an unconscious desire to abort or an emotional unpreparedness for pregnancy [83]. Medical dismissal can contribute to distress, stigma, distrust in the medical system, and reluctance to seek care [84–86]. The discovery of GDF15 as a hormonal trigger, and that NVP sensitivity has a large genetic component, together provide a strong legitimizing effect to women suffering from pregnancy sickness: it is a molecularly tractable, and possibly treatable, medical condition.

Pregnancy sickness involves both innate aversive responses such as generalized nausea, anorexia, and heightened olfactory sensitivity, as well as targeted and experience-dependent responses such as conditioned aversion to specific foods. These two forms of aversions have distinct developmental underpinnings, but both are regulated by GDF15. Circulating toxins induce nausea irrespective of visual or olfactory stimuli, as in chemotherapy-induced nausea, which involves GDF15 [87]. Specific food triggers that provoke aversion can also develop through associative learning [88]. This learning process, too, is influenced by GDF15 [89]. While targeted food aversions are reported during the 1^st^ trimester of pregnancy, they are in the majority of cases extinguished after delivery, not committed to long-term memory [90].

GDF15’s dual developmental roles in both nonspecific aversions and conditioned taste aversions may explain why both can occur in pregnancy, but conditioned taste aversions in pregnancy are likely maladaptive or neutral. Learned aversions are only beneficial if the stimulus is paired with an actual deleterious event, not just placental hormones. Without a way for the mother to receive accurate feedback about real teratogenic risks, acquired distastes due to unfortunate pairings of tastes or smells with episodes of pregnancy nausea are unlikely to be functional. For this reason, the majority of evolutionary discussions of pregnancy sickness have focused instead on innate, not learned, food aversions [10, 14].

With substantial variation across individuals and even between pregnancies in the same woman, pregnancy sickness shows phenotypic plasticity, or the capacity for a single genotype to lead to different phenotypes across environments. A substantial genetic component is undeniable: twin studies from Finland and Spain estimate a heritability of NVP over 70% [91, 92]. Hyperemesis gravidarum, too, shows familial aggregation indicative of genetic causation [93]. Genome-wide association studies have recovered risk variants near biologically plausible loci, replicated across European and Japanese cohorts [23, 51]. This maternal background likely interacts with environmental and fetal genetic factors to determine how pregnancy sickness ultimately manifests—pointing towards genotype-by-genotype-by-environment causation (Box 1). Maternal genetic variants around the *GDF15* locus influence baseline sensitivity to emetogenic signals from the placenta by lowering pre-pregnancy circulating levels of GDF15 [22, 23]. Fetal genotype determines the magnitude of placental GDF15 output, which also shows a quantitative association with the severity of NVP [22]. Meanwhile, the response to pregnancy hormones is modulated by environmental factors like smoke and toxin exposure, as well as maternal condition, including infection and nutritional status, certain conditions like beta thalassemia, physical activity, and psychological state.

**Box 1: Is NVP heritable, variable, and does it contribute to fitness?**

For a trait to be subject to continued natural selection, it must be (1) heritable, (2) vary across individuals, and (3) have a non-zero correlation with reproductive success [218].

With respect to (1), NVP has significant heritability. A twin study of 1723 women from Australia, Finland, Spain, the UK and Denmark quantified maternal heritability values for presence/absence of NVP of 73% (95% CI 57–84), for duration 51% (95% CI 36–63), and for severity 53% (95% CI 38–65) [91, 92]. It is also influenced by fetal genes affecting GDF15 output, and thus has paternal heritability, although the fetal genotype is estimated to contribute less to heritable NVP risk than maternal [22, 50].

With respect to (2), NVP severity varies considerably between individuals, as well as between global populations. Phenotypic variation is considerable: at any given month of pregnancy, the severity of NVP among individuals follows a right-skewed distribution, with a proportion of individuals unaffected and a tail of those with high severity [219]. This variation is partially underlain by maternal genetic differences influencing the sensitivity to GDF15 challenge [102]. Genes implicated in maternal sensitivity include *IGFBP7*, *PGR*, *FSHB, IGSF11,* and *TCF7L2* [50]. Risk alleles are not rare: variants in 19p13.11, a region including *GDF15*, and 11q22.1, a region including *PGR* and *TRPC6*, were identified in both European and Japanese cohorts with minor allele frequencies of ~20%–25% in the European populations studied and ~ 45%–50% in the Japanese populations [23, 51]. Additional variants near *PGPEP1* associated with circulating GDF15 levels were carried by approximately 45%–50% of a Japanese cohort [51, 220]. Fetal genetic variants with positive effects on *GDF15* production also contribute [50]. Remaining variation in NVP is likely explained by non-genetic factors, including pre-pregnancy exposure to toxins, infection, and psychosocial risk factors. Beta thalassemia and pre-pregnancy maternal smoking are both negatively associated with HG risk, whereas psychiatric conditions including anxiety and depression are positively associated [22, 130, 206]. Together, these findings suggest that variation in NVP severity is driven by genotype-by-genotype-by-environment causation (Box 1  **Figure**).

As for correlation with reproductive success (3), GDF15 is not essential for pregnancy, and genotyping has identified at least 8 individuals homozygous for *GDF15* loss-of-function and more than 200 heterozygotes with no evidence of infertility or overt disease [31]. Nevertheless, several studies in American hospitals have reported associations between NVP and reduced incidence of pregnancy loss. A study of 873 pregnancies found that mothers who reported vomiting during the first 20 weeks had an odds ratio of miscarriage of 0.18 (95% CI: 0.06–0.53) [221], another study of 414 pregnant women reported an association between vomiting and a reduced rate of fetal death (OR = 0.24) [222], and a 2010 study of 2407 women found an odds ratio of 0.31 (95% CI: 0.23–0.42) for pregnancy loss in women with vomiting [223]. A prospective study following 979 women from conception showed that those with nausea and vomiting had a 75% lower hazard of miscarriage at any point during pregnancy [224].

While these studies point consistently in the same direction, the cause of observed associations with pregnancy failure is contested. Some have argued that it is merely reverse causation or confounding—inherently low-viability embryos both produce lower levels of GDF15 and are more prone to failure [16, 209]. It is unlikely that differential exposure to dietary toxins due to maternal aversions are the cause, as many known reprotoxins, like endocrine-disruptive chemicals, cannot be actively avoided by food preference changes, or are avoided by rational decision-making beyond what is realistically attributable to hormones [225]. A more likely pathway for small fitness effects would be through effects on implantation success, placental development, or immune regulation. Larger-scale epidemiological investigation will be required to rule out alternative explanations.

Hyperemesis gravidarum is unconditionally deleterious. Women with HG show a miscarriage rate of more than double of those without, have serious morbidities related to excessive vomiting and dehydration, and their offspring are at greater risk of cardiovascular, neurological, and immune pathologies [226, 227].

The plasticity apparent in NVP and its more extreme forms is the signature of an evolved, and fallible, system of behavioral defense subject to environment-dependent regulation. Before attributing adaptive function to pregnancy sickness, it is necessary to first define the broader behavioral defense system pregnancy sickness is a part of.

**Box 1:**
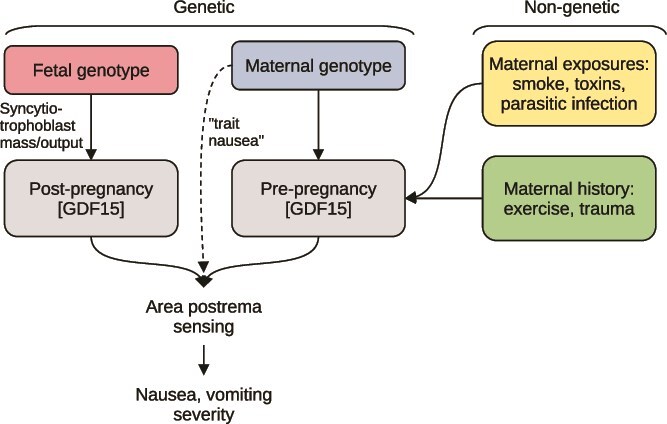
Figure. Genotype-by-genotype-by-environment model of pregnancy sickness. NVP severity depends upon a comparison of post-pregnancy GDF15 levels to pre-pregnancy levels, both of which have genetic and environmental influences. The thresholds at which symptoms result given the same change is further tuned by maternal sensitivity (hashed arrow labeled “trait nausea”).

## REVISING THE NULL HYPOTHESIS FOR PREGNANCY SICKNESS

Margie Profet, who popularized the hypothesis that NVP functions to avoid toxin exposure, described her analytical style as adaptationist [12, 94], citing George Williams’s *Adaptation and Natural Selection* [95]*.* Adaptationist analysis seeks to understand biological traits through functional or design-based reasoning by asking, what selective advantage might the trait confer? Evolutionary medicine has often explicitly utilized this framework [96]. A lacuna in adaptationist analysis is that it frequently assesses individual elements of the organism’s phenotype independently as a simplifying assumption, without considering their developmental, genetic, or functional integration with other traits. Incorrect or overzealous atomization can lead one to propose adaptive functions for parts of an organism which are simply strung along by linked traits undergoing evolution, or are necessary byproducts of developmental or physiological processes [97]. The classic example is the human chin: early theories for the chin as a sexual signal were made obsolete by a more parsimonious developmental explanation that the chin is a byproduct of the coming together of alveolar and mandibular growth fields. Neuroendocrine systems, too, are composed of modular subunits that are executed together, and evolve together [98, 99]. Are functional interpretations of individual components of pregnancy sickness, such as nausea or food aversion, vulnerable to the same fallacy? Avoiding this pitfall requires first defining the underlying character which can be subject to natural selection [100, 101].

In pregnancy, nausea and vomiting occur as part of a larger syndrome [102]. Like the chin, nausea is only the tip of a larger iceberg. The set of correlated symptoms behind pregnancy sickness is known by different names in different fields. It has been described in medical literature as sickness behavior [103] or sickness syndrome [104], in psychological literature as the behavioral immune system [105], and in immunobiology and physiology as the food quality control system [25].

**Figure 2:**
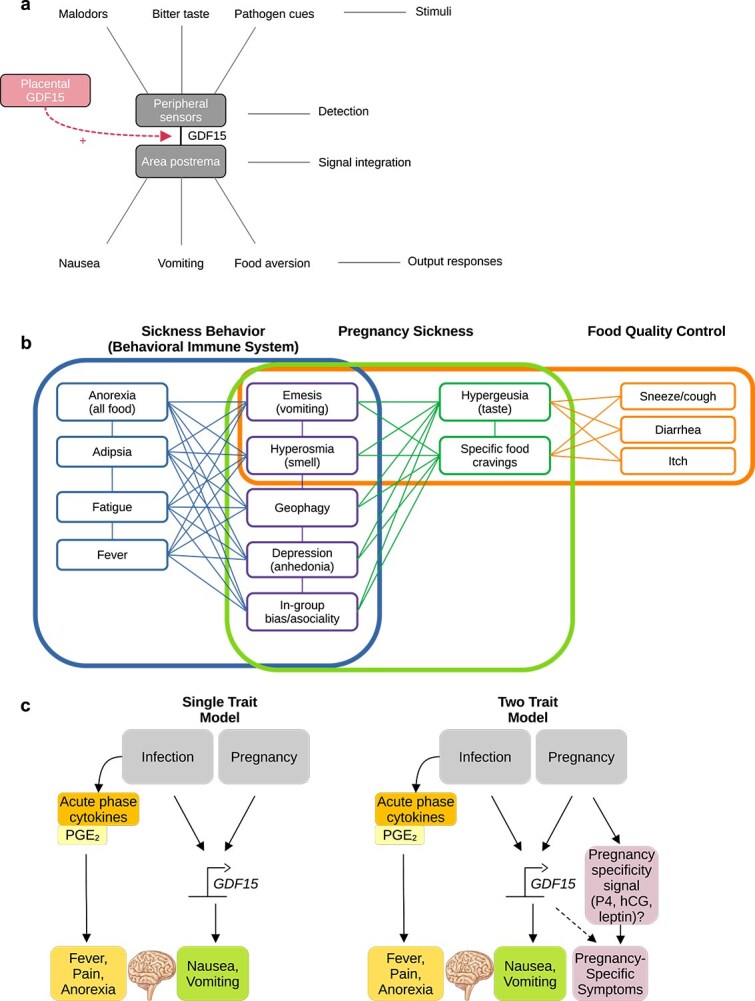
Relationship of pregnancy sickness to facultative sickness behavior in response to infection and toxin exposure. (a) Model of the area postrema-mediated system of defense against food-borne dangers. Placental production of GDF15 stimulates the area postrema, leading to nausea and vomiting in the absence of normal stimuli. (b) Venn diagram of symptoms of pregnancy sickness in comparison to two leading frameworks for behavioral defenses, sickness behavior (the behavioral immune system) and food quality control. If pregnancy sickness has unique adaptations absent in non-pregnancy-related behavioral defenses, a co-occurrence network of these symptoms should show a modular structure. (c, d) Comparison of models of NVP as an elevated state of sickness behavior (single trait model, c) or a distinct neuroendocrine trait (two trait model, d). P4: Progesterone.

These models share a similar architecture: dangerous stimuli such as noxious foods, odors, or pathogens are neurologically or chemically sensed, integrated with signals from host physiology, and behavioral defenses are elicited (Fig. 2a). Sickness behaviors refer to the stereotyped behaviors a human patient or other animal displays when infected by a pathogen, including nausea, anorexia, malaise, and some signs of depression [103]. Infection-induced cytokines such as IL-6, IL-1β, TNF, and prostaglandin E_2_ contribute to anorexia, fever, and increased sensitivity to pain (hyperalgesia) [104, 106, 107], and activate the second messenger NF-KB which stimulates GDF15 production [108]. The behavioral immune system model expands upon sickness behaviors by proposing that a complementary set of anticipatory behaviors mediated by the emotion of disgust, in both visceral and more abstract forms, mitigate disease risk before it happens [105, 109–111]. These proactive defenses include innate visual, olfactory, and gustatory avoidance of dangers such as toxins, phytochemicals, and bodily waste products [112], as well as quarantining behaviors like social withdrawal and xenophobia [113, 114]. While the behavioral immune system is primarily cognitive, the food quality control system model is more physiological, neurological and immunological. It proposes that the digestive, nervous, and immune systems cooperate to sense and defend against toxins. Potentially harmful foodborne substances are detected via proxies like smell or appearance, by TAS2R bitter taste receptors which trigger GDF15 production in response to alkaloids [43], or by intestinal epithelial and immune cells [26, 115].

Only some of the defensive behaviors established in these models are routinely activated during pregnancy (Fig. 2b). This shared core includes nausea and vomiting, food aversions, geophagy, and possibly depression-like symptoms. Depression is the non-food-related sickness behavior most reliably associated with NVP. Evidence from a Dutch cohort suggests that women with more severe NVP show more severe symptoms of depression and anhedonia [116]. Geophagy, or the consumption of non-nutritive substances such as dirt or clay which is a key component of animal sickness behavior, manifests as pica cravings reported in the first trimester of human pregnancy [117, 118]. Geophagy is elicited via the same GDF15-dependent neuroendocrine pathways that drives nausea and vomiting [119]; in rodents, which cannot vomit, GDF15 induces geophagy instead. On the other hand, pregnancy does not induce hallmark inflammatory features such as fever, redness, pain, adipsia, and whole-body malaise [103]. Nor does pregnancy typically cause the indiscriminate anorexia seen in infection: cytokine-induced anorexia persists in *Gdf15* and *Gfral* null mice, indicating that it acts through GDF15-independent pathways [120].

The behavioral immune system model predicts that more abstract aversions, including sensitivity to contagion cues, sexual or moral disgust, and xenophobia should be heightened in pregnancy, but these are not all empirically observed. Surveys from the USA and Poland found that aversions towards foods and bodily fluids are heightened in the first trimester, but no aversions to cues of death, injury, non-normative sexual behavior, or sociomoral transgressions were observed [121, 122]. A widely cited cross-sectional study of U.S. women reported elevated ethnocentric sentiments during the first trimester [123], but no direct association with nausea severity or plausible origin in placental GDF15 has been shown.

These patterns suggest that placental GDF15 selectively triggers food-related nausea and aversions rather than the entire suite of sickness behaviors and behavioral-immune defenses (Fig. 2c). Systems biology tools such as co-occurrence network analysis [124] will be necessary to more quantitatively explore the relationship between pregnancy sickness and other sickness behaviors. Correlated symptoms with NVP may also be culturally conditioned or influenced, and require sampling of diverse global populations. Nevertheless, cursory comparison suggests that pregnancy sickness is not an evolutionarily novel behavior, but a targeted subset of sickness behavior induced by pregnancy hormones.

Hyperemesis gravidarum is comorbid with hypersensitivity disorders of food quality control and behavioral immunity. Hyperactive food quality control has been proposed to underlie allergy [25, 125, 126]. While typical allergic defenses, like cough, sneeze, itch, and diarrhea are not prominent in pregnancy, allergy does significantly predispose to severe NVP. In a U.S. cohort, allergy was found to more than double the odds of prolonged versus short-duration HG (OR = 2.12; 95% CI: 1.17–3.91), emerging as the top predictor of duration of the disease [127], and a later study reported significantly higher prevalence of allergy in HG patients compared to controls (OR = 1.61; 95% CI: 1.22–2.11) [128]. Within behavioral defenses, anxiety, depression, and obsessive-compulsive disorder have been theorized to be pathological exaggerations of adaptive defense behaviors [129]. A Danish study found that women with a history of depression were more likely to have severe NVP (OR = 1.53; 95% CI: 1.11–2.11) [116], and studies at Turkish hospitals reported that women with hyperemesis gravidarum had higher rates of major depression (OR = 3.91; 95% CI: 1.12–13.70), generalized anxiety disorder (OR = 5.27; 95% CI: 1.33–20.86), and obsessive-compulsive disorder (OR = 4.51; 95% CI: 1.11–18.29) [130], as well as somatosensory amplification, a disorder causing benign sensations to be perceived as noxious [131], but these results showed unusually high variation. If replicated, these comorbidities would suggest that trophoblast-derived signals such as GDF15 engage a broader defense architecture, and that disorders which lower activation thresholds of other systems may render women more vulnerable to severe pregnancy sickness.

Dietary changes in pregnancy depart from canonical sickness behavior in their specificity. Pregnant women across cultures report positive food cravings for dairy, sweets, starches, and fruits [10], and aversions specifically to meats, alcohol, coffee, and other substances [132–134]. Such specific cravings and aversions are unlikely to arise from GDF15 alone. Wang and Medzhitov [135] have suggested that a common cachectic signal like GDF15 may combinatorially associate with context-specific cues such as lipopolysaccharide or viral products to specify context-specific bacterial or viral variants of sickness behaviors. Context-specific hormones such as progesterone, hCG, or leptin would be plausible candidates to fine-tune the behavioral response in pregnancy (Fig. 2d).

Research into the GDF15-GFRAL/RET signaling axis has revealed that nausea and behavioral toxin defenses are ancient, likely predating the origin of mammals. The true evolutionary novelty of pregnancy sickness therefore lies not in these behaviors themselves, but in their endocrine activation by the placenta. This raises the bar for evolutionary theories of NVP. A revised null hypothesis for the evolution of pregnancy sickness must be that it is nothing more than endocrine activation of a pre-existing system for nausea and vomiting by placental hormones, requiring perhaps no more than a change in promoter to the *GDF15* gene to evolve. The alternative hypothesis—that pregnancy sickness involved the acquisition additional mechanisms to determine which foods are potentially teratogenic, or pregnancy-specific metabolic circuits—bears the burden of proof. For the time being, adopting the null hypothesis of placental GDF15 production as the sole enabling trait renders the trait simple enough for its phylogenetic origin to be resolved.

## PHYLOGENETIC ORIGIN

NVP was historically assumed to be human-specific, as it could only be reliably identified by self-report [14, 136]. Discovery of GDF15 as a causal hormone of NVP casts doubt on this assumption. Unlike hCG, GDF15 is widely conserved across mammals, as is the neuroendocrine circuit for sickness behavior. Linkage of NVP to this quantifiable proxy allows more rigorous testing of its phylogenetic breadth.

### Placental GDF15 production rose to prodigious levels uniquely in catarrhine primates

The broadest published comparison of serum GDF15 levels assessed two primates and two rodents, finding that humans and rhesus macaques show similar levels of circulating GDF15 during pregnancy, while levels in mice and rats are minimal [137]. The cis-regulatory region of primate *GDF15* diverged after their divergence from rodents, with primates having a unique enhancer region (Ulirsch et al., 2014), but the specific elements driving *GDF15* expression in the placenta remain unknown.

**Figure 3:**
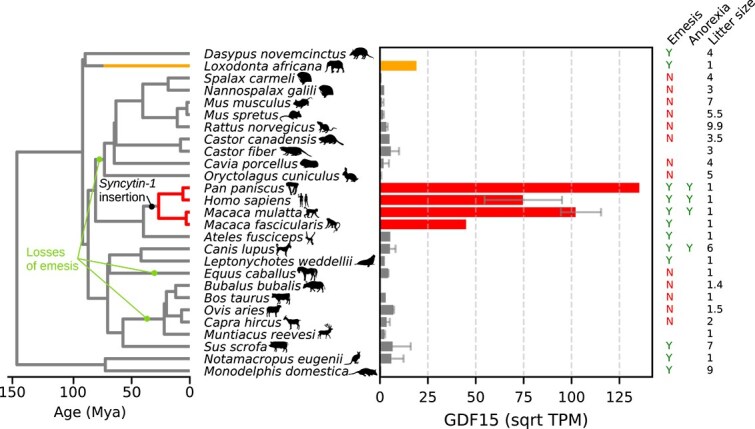
Extreme placental GDF15 production evolved in catarrhine primates. Whole-placental *GDF15* mRNA expression (square-root transcripts per million) is plotted against a phylogeny (left). Transitions to arbitrary thresholds of moderate expression (200 TPM) and high expression (1000 TPM) are indicated on the phylogeny. Branches along which the capacity for emesis were lost are marked with arrows. Genomic insertion of three viral elements (*ERVW-1, ERVH48–1, and ERV3–1*) essential for catarrhine-specific syncytiotrophoblast development is marked as “Syncytin-1 insertion”. Presence of emesis and reports of anorexia during pregnancy are plotted as presence (Y), absence (N), or missing data (blank) for each species. Expression data are compiled from published datasets on the NCBI gene expression omnibus (citations in Table S1). Tree topology and divergence dates are from TimeTree 5 [147], and litter size values are from AnAge [148].

RNA-sequencing data from all 27 species with available placental sequencing data in the NCBI Gene Expression Omnibus [138–146] show a clear phylogenetic pattern: extreme placental levels of *GDF15*, two to three orders of magnitude greater than rodents, are present only in catarrhine primates (Fig. 3). Catarrhini consists of apes (Hominoidea) including humans and bonobos, and Cercopithecoidea, including the crab-eating macaque and rhesus macaque, all four of which have highly elevated placental *GDF15*. In contrast, the black-headed spider monkey *Ateles fusciceps*, a member of the sister group to Catarrhini, has low levels of placental *GDF15* (Fig. 3). The presence of highly elevated circulating GDF15 levels during pregnancy is therefore phylogenetically restricted, but not human-specific.

Curiously, the lineage showing the second-greatest level of placental *GDF15* outside catarrhines is the African elephant, evolutionarily distant from Old World primates but sharing with humans life history traits such as a singleton birth and high investment in offspring. Whether placental GDF15 drives physiological or behavioral changes in elephants, and whether they exhibit a convergent form of pregnancy sickness, deserves further investigation.

This positioning allows certain phylogenetic explanations to be ruled out. The evolutionary loss of emesis in rodents and ruminants [149–153] does not explain their lack of pregnancy sickness, as several mammals retain the ability to vomit but lack placental *GDF15* production (Fig. 3). Furthermore, anorexia reported in pregnant macaques [154], marmosets [155], dogs [156], and even snakes, chickens, and octopuses [30] has been proposed as homologous to human pregnancy sickness, but this does not agree with the observed restriction of placental *GDF15* expression to Catarrhini. As reports of anorexia during pregnancy are most often anecdotal, it is possible that they would not hold up to rigorous investigation, or alternatively, anorexia could be elicited independently of GDF15 such as through the melanocortin system [157]. Reports of pregnancy-associated anorexia may reflect common life history principles, but not common cause, with human pregnancy sickness.

### Syncytiotrophoblast evolution explains the origin of placental GDF15 better than diet

Catarrhine primates, who share placental *GDF15* production, diverged from New World monkeys around 43 million years ago, placing the likely origin of pregnancy sickness in the Eocene [147]. Profet’s [12, 14] prophylactic hypothesis situates the origin of NVP in human foragers in the late Pliocene or Early Pleistocene only 2–3 million years ago, where it would have promoted avoidance of dietary teratogens. Its adaptive benefit is proposed to have been greatly enhanced after the invention of cooking, which expanded the range of dietary plants [12], and a transition to ‘experimental omnivory’ [14, 29], or the sampling of diverse and potentially toxic foods concomitant with safeguards like food neophobia and innate taste aversions [158]. This phylogenetic explanation is incompatible with an origin in stem Catarrhini. Catarrhine primates underwent no shift to an exploratory feeding strategy, and meta-analysis of dietary preferences across 155 primate field studies reveals no consistent pattern of plant secondary metabolite avoidance that is unique to catarrhines, only moderate tannin avoidance in some leaf-eating colobines that is absent in humans and other apes [159]. Thus, the origin of pregnancy sickness coincides with no known dietary shift or novel avoidance behavior common only to species with the trait.

In contrast, what members of Catarrhini do share is a suite of derived placental traits related to the syncytiotrophoblast cell type which is the source of GDF15 and other hormones (Fig. 1). These begin with multiple germline incorporations of retroviral fusogens that enable syncytiotrophoblast cell development [160]. An initial acquisition of *Syncytin-2* (*ERVFRD-1*) in stem primates [161] was followed by catarrhine-specific insertions of three more elements, *Synyctin-1* (*ERVW-1*), necessary for syncytiotrophoblast maturation [162], *Suppressyn* (*ERVH48–1*), active in primate early-fusing syncytiotrophoblast [163], and *ERV3–1*, active in early syncytiotrophoblast [164] (Fig. 3). These genomic innovations coincided with changes to the hormones the syncytiotrophoblast produces: catarrhines produce placental leptin at levels around 10 times higher than platyrrhines [165], and acquired mutations to the β subunit of hCG which increased its circulating half-life three- to four-fold [166]. Anatomically, catarrhine-specific syncytiotrophoblast innovations manifest as an invasive (hemochorial) placenta with a cytotrophoblast shell and elaborately intertwined villous structure that is lacking in New World monkeys, and which penetrates maternal tissues more deeply, to the myometrium, in contrast to trophoblast invasion in New World monkeys stops at the endometrium [167, 168]. This suite of placental adaptations in the catarrhine stem lineage provides a more coherent explanation for the phylogenetic emergence of NVP than a shift in diet.

## ADAPTIVE FUNCTION

**Figure 4:**
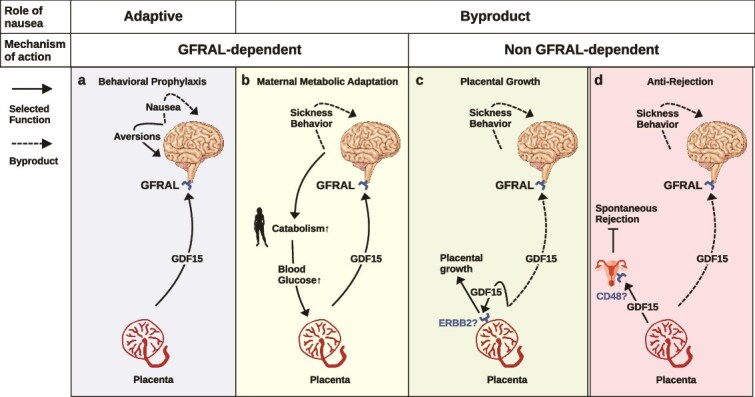
Evolutionary hypotheses for placental GDF15 production. Functions which were directly selected for are marked with continuous arrows, while byproduct effects are marked by dashed arrows. (a) The behavioral prophylaxis hypothesis [10, 13, 14] proposes that behavioral changes due to NVP are directly selected for due to avoidance of ingesting environmental toxins. (b) The maternal metabolic adaptation hypothesis [17, 169] holds that GDF15’s effects on the brain are selected for due to downstream induction of catabolism in maternal tissues which increase blood glucose. Nausea is merely a means to this end. (c) The placental growth and development hypothesis holds that GDF15 has local effects on placental growth and that all maternal effects, including NVP, are a byproduct. (d) The anti-rejection or embryo selection hypothesis [15, 16] predicts that the causative hormone of NVP has local effects on the endometrium that determine whether pregnancy continues. Excessive production of emetogenic hormones is either necessary or strategically advantageous to pass maternal quality control.

The final level of analysis is that of adaptive function. Several theories have been advanced for the original selective advantage of pregnancy sickness. In some nausea is the selected trait, and in others it is a byproduct of selection on other physiological features (Fig. 4).

### Behavioral prophylaxis hypothesis

The oldest and most persistent is the prophylactic hypothesis, which holds that pregnancy sickness facilitates behavioral avoidance of noxious foods, such as meat-borne pathogens and plant secondary metabolites [10, 13, 14, 170]. The theory has also been referred to as ‘compensatory prophylaxis’ to foreground the assertion that pregnant females require behavioral defenses because they are inherently vulnerable to infection from reproductive immunosuppression [121]. However, as the assumption that pregnancy requires immunosuppression has been contested [171, 172], only the prophylactic function will be considered here.

In the prophylactic model, behavioral changes due to NVP which lead to targeted food aversions are directly selected for, with nausea as a means to this end (Fig. 4a). Functional fittedness of NVP to teratogen avoidance is supported by a striking temporal correlation between mean symptom severity and the development of key organ systems in the embryo, when teratogen exposure could lead to fetal demise [10]. In the original framing, Profet [14] hypothesized that pregnancy sickness is a maternal adaptation which co-opted an incidental fetal hormone (hCG) as a cue to trigger nausea, while the fetal hormone itself was not inherently emetogenic. Discovery of the role of placental GDF15 requires modification this claim: as GDF15 induces nausea, anorexia, and food aversions in wide range of contexts [87, 89], no specific maternal response needed to evolve for pregnancy. Many of the apparently functionally fitted teratogen-avoidance behaviors attributed to pregnancy sickness instead come ‘for free’ with GDF15 production. Profet noted that the key deciding factor of whether pregnancy sickness is an adaptation is whether ‘it achieves an adaptive event with sufficient precision, economy, efficiency, and complexity to indicate functional design’ [12]. Discovery of the GDF15 mechanism and of non-pregnant behavioral forms of toxin avoidance, suggest that pregnancy sickness has surprisingly less complexity—but much more economy and efficiency—than previously believed.

Some versions of the prophylactic hypothesis posit an adaptive function for geophagy in pregnancy [12]. Ingestion of dirt and clay can protect the integrity of gut mucosa and inhibit bacterial growth [173], which make geophagy an adaptive sickness behavior in many animals. However, benefits of geophagy specifically during pregnancy have not been demonstrated. Introduction of 20% kaolin clay to the diet of pregnant rats led to ~10% reduced fetal birthweights and maternal anemia [174], although human studies have failed to detect an effect on birth weight [175]. Clay consumption has been linked to increased levels of heavy metals such as lead, arsenic, and cadmium in blood serum and reduced calcium and potassium [176]. These observations make prophylaxis via geophagy an unconvincing functional explanation.

There are several weaknesses to the prophylactic hypothesis. First, the desensitization property of GDF15 sensitivity [22] appears to be a fundamental design flaw if nausea itself during pregnancy is an adaptation: it is precisely individuals living in environments with high exposure to toxins and pathogens who would need it most, and yet would be the least likely to experience NVP due to presumably high pre-pregnancy levels. Furthermore, pregnancy-specific mechanisms to detect and avoid plants high in toxic secondary compounds are lacking in ruminant herbivores, such as sheep and goats, even though they have high environmental exposure to plant secondary compounds [177]. While counterfactual questions are generally frowned upon in evolution [178], an obvious one in challenge of the prophylactic model is why, if dietary exposure and fetal vulnerability defines the selective regime, more taxa outside of Catarrhini with similar or higher dietary risk have not evolved placental GDF15 production.

### Maternal metabolic rewiring hypothesis

Alternatively, it has been suggested that reduced maternal nutrient intake in the 1^st^ trimester rewires maternal metabolism in a way that benefits fetal growth (Fig. 4b). Reduced food intake in early pregnancy is associated with lower maternal insulin and insulin-like growth factor 1, which have been suggested to indirectly benefit fetal growth by driving maternal anabolic processes [169]. Crespi [17] revised this hypothesis to propose that GDF15’s anorectic effects on the brain via binding GFRAL/RET induce catabolism in maternal tissues that increase blood glucose, in turn facilitating nutrient transfer to the placenta by mobilizing glucose stores. Maternal starvation is indeed associated with greater placental growth: in the 1944–1945 Dutch famine, women who either conceived or were in the 1^st^ trimester during the onset of the famine gave birth to offspring with larger placental weights, whereas those who were later in pregnancy at the onset of the famine did not [179]. However, this benefit to placental growth does not necessary carry over to the embryo. The birthweights of babies affected by the Dutch famine showed no difference between the two groups, and without a fetal effect it is not clear that starvation can have a direct positive effect on offspring fitness.

### Placental growth and development hypothesis

Another functional explanation advanced for NVP is that GDF15’s primary function is to locally drive placental growth and invasion, while systemic effects on maternal behavior, including NVP, are incidental byproducts of its passage into the bloodstream (Fig. 4c). Trophoblast cells treated with exogenous GDF15 display enhanced cell migration and increased implantation success in an *in vitro* model [180], as well as reduced invasion capacity after *GDF15* knockdown [181]. Abnormally low levels of circulating GDF15 have also been associated with preeclampsia, a disease involving insufficient trophoblast invasion [182]. These effects resemble GDF15's reported role in cancer, where elevated levels and *GDF15*-activating mutations are associated with tumor cell proliferation and metastasis [183]. Trophoblast and cancer are both proliferative and invasive stem-like tissues, and placental GDF15 production could be the consequence of a positive-feedback loop which the embryo and the mother have not managed to counteract (Box 2).

**Box 2: Does GDF15’s role in cancer mirror its function in trophoblast?**

Tumors develop by an evolutionary process known as clonal selection, acquiring mutations which confer proliferative and metastatic abilities [228, 229]. Analysis of data from The Cancer Genome Atlas has revealed an association between *GDF15* expression levels and metastatic and immune phenotypes associated with worse prognosis [183]. A productive way to interpret parallels between the evolution of placental *GDF15* expression in catarrhine primates and tumor *GDF15* mutation in disease is as convergent adaptations [230]—the former on the macroevolutionary scale, and the latter microevolutionary, occurring repeatedly during clonal selection of tumors within individual hosts. It is unlikely that the fitness advantage afforded to cancer cells by GDF15 production is due to dietary changes by the host. Instead, the following explanations are possible:

1. GDF15 confers a non-GFRAL-mediated boost to proliferation and/or migration ability of tumor cells which is convergently adaptive in invasive trophoblast.
2. GDF15 promotes immune evasion through non-GFRAL-mediated pathways. The mechanisms by which the placenta promotes immunotolerance in species with invasive pregnancy utilizes some of the same mechanisms as tumors [172, 231].
3. GDF15 acting through GFRAL-RET promotes host cachexia, wasting, and catabolism which increases free nutrients available to the tumor, and therefore functions indirectly as a driving mutation.
4. GDF15 is a stress-induced factor triggered by hypoxia or replicative stress in the tumor microenvironment. It is a passenger rather than a driver of malignancy. The *GDF15* promoter contains a p53 response element which drives expression under conditions of cellular stress [232, 233]. More aggressive tumors are subject to more cellular stress, leading to a correlation of GDF15 levels with malignancy which is unrelated to the biochemical functions of GDF15.
5. Cancer cells activate early trophectoderm gene-regulatory programs as part of a de-differentiation process that allows them to access a more stem-like state [234, 235]. In catarrhines, this state includes syncytiotrophoblast products like GDF15 and β-hCG as a consequence of trophoblast gene-regulatory evolution. Association with malignancy is a byproduct of this gene-regulatory network structure rather than functionally advantageous.

The first three of these explanations for GDF15’s role in cancer malignancy are related to the functional theories for NVP, with (1) corresponding to the autocrine growth hypothesis, (2) supporting the anti-rejection hypothesis, and (3) supporting the maternal metabolic rewiring hypothesis (see Fig. 4). Explanations (4) and (5) would mean that the trophoblast-tumor analogy with respect to GDF15 is not functionally informative.

Such an effect, however, would require a non-canonical receptor mechanism. Extravillous trophoblast and syncytiotrophoblast cells express high levels of one of the proposed non-canonical receptors for GDF15, ERBB2 [69, 79, 184]. ERBB2 lacks a direct ligand-binding domain, but has been shown to bind GDF15 as part of a larger, incompletely characterized protein complex likely containing TGF-β family members [185]. Thus, GDF15 binding to a multimeric receptor in trophoblast cells may enhance placental development, but molecular details have yet to be elucidated.

### Anti-rejection hypothesis

Finally, the anti-rejection or embryo quality hypothesis [15, 16] proposes that the hormone driving NVP locally modulates the endometrium to promote embryo survival (Fig. 4d). Under this hypothesis, the causative hormone reflects embryo viability: more viable embryos secrete more hormones, including hCG and GDF15, to promote uterine retention and tolerance, while lower-quality embryos that fail to inhibit spontaneous abortion are rejected [15, 186]. This reverses the causal story compared to the prophylactic hypothesis. Instead of the prophylactic model of:


\begin{align*} \textrm{ embryo hormone production }\ &\rightarrow\ \textrm{ maternal toxin avoidance } \\&\rightarrow\ \textrm{ embryo survival } \end{align*}

the anti-rejection hypothesis posits a chain from:


\begin{align*} \textrm{ embryo quality } &\rightarrow\ \textrm{ elevated hormone production }\\ &\rightarrow\ \textrm{ reduced uterine rejection } \end{align*}

with maternal nausea as a byproduct (Fig. 4d).

An embryo could plausibly influence its own acceptance through tolerogenic immune pathways, or pro-gestational hormone production. The former is more likely to be performed by GDF15, and the latter by hCG. GDF15 promotes M2 (anti-inflammatory) macrophage polarization when paired with IL-4 and IL-15 [187], and mediates anti-inflammatory drug effects in peritonitis models [188]. In cancer, higher GDF15 levels correlate with T cell exclusion from tumor tissue, leading to the proposal that it functions as a ‘T cell repellent’ [189]. Neutralizing antibodies against GDF15 enhance CD8^+^ T-cell infiltration and improve anti-PD-1 immunotherapy efficacy in both mice and humans [183, 189], suggesting an anti-immune-surveillance effect. In pregnancy, recombinant GDF15 administration has been shown to reduce embryo resorption in mouse models of infection-induced resorption [181], although the study used recombinant human GDF15 synthesized in mammalian cells, requiring confirmation to exclude the possibility of TGF-β contamination [80].

An effect on spontaneous pregnancy loss implies that GDF15 primarily acts locally on the uterus via GFRAL-independent pathways. One possible binding partner is CD48 on the surface of regulatory T cells. GDF15 has been shown to interact with T cell CD48 to induce the anti-inflammatory regulatory T cell fate by stabilization of the transcription factor FOXP3 [78]. These cells (CD4^+^ CD25^hi^ FOXP3^+^) function in pregnancy to suppress fetal allorejection [190]. Research on immunomodulatory effects of GDF15 is rapidly progressing, but the absence of a biochemically plausible receptor remains a major obstacle to a purported role in pregnancy.

Overall, it is plausible that embryonic hCG indeed evolved as an anti-rejection signal as previous theories have alleged [16]; however, hCG is now known not to be the proximate cause of pregnancy sickness. GDF15 does have demonstrated immunomodulatory properties that suggest that it could play a similar role at the fetal-maternal interface to suppress immune rejection. However, more research is needed specifically in a pregnancy context [181]. Any effect on embryo survival must not be absolute, as *GDF15* loss-of-function mutations have no demonstrated effect on human fertility [31].

## NATURAL SELECTION AND PATHOLOGICAL PREGNANCY SICKNESS (HYPEREMESIS GRAVIDARUM)

In addition to the adaptive function of normal-range pregnancy sickness, there lies another question: why does the risk of hyperemesis gravidarum persist, despite it endangering reproductive success and survival of both the mother and fetus? Here, I outline three reasons why this pathology has not been eliminated by natural selection.

### Hyperemesis gravidarum risk is maintained by selection for toxin avoidance and infection tolerance

**Figure 5:**
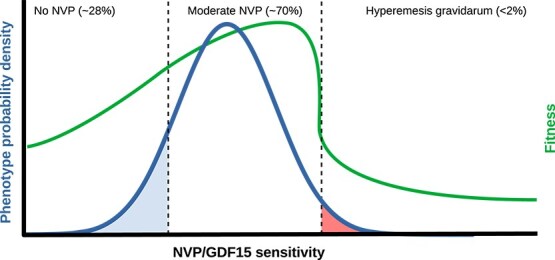
Cliff edge model for hyperemesis gravidarum risk in modern populations. Putative fitness distribution of NVP/GDF15 sensitivity during the first trimester shows a gradual increase until the threshold for triggering of hyperemesis gravidarum, at which point the fitness function declines. Assuming that the population phenotype distribution must be normal, the distribution maximizing mean fitness includes both unaffected individuals (left shaded region) and pathologically affected individuals (right shaded region). Individuals with maladaptive extreme phenotypes correspond to the approximately 1%–2% of human pregnancies with hyperemesis gravidarum. After Mitteroecker and Merola [191].

One explanation for hyperemesis gravidarum is that it represents pathological hypersensitivity of a system of defense which is adaptive in normal range. Consider that the responsiveness of this system is determined by a hypothetical quantitative trait of ‘GDF15 sensitivity’ (Fig. 5). Sensitivity may be determined by receptor abundance, signal transduction efficiency, or other factors. GDF15 sensitivity benefits host tolerance during infection [44], and enough GDF15 sensitivity to induce NVP shows a statistical association with reduced risk of miscarriage or fetal demise (summarized in Box 1). As with other defense systems, natural selection favors high sensitivity to avoid false negatives, increasing the risk of pathological false positives [192].

The optimal level of GDF15 sensitivity differs between individuals living in high- and low-pathogen environments, as well as between males and females. A gene with pleiotropic effects on both infection tolerance and pregnancy sickness cannot be simultaneously optimized for all contexts. Any allele that modulates GDF15 sensitivity will spend approximately half of its time in male genomes, half in female genomes, and only a small fraction of its time in genotypes that result in hyperemesis gravidarum. It will spend a much larger fraction of time in genotypes where it confers a modest benefit without inducing severe pregnancy complications. Consequently, risk-increasing alleles can be under positive, rather than negative, selection [193]. This has all the makings of an evolutionary trade-off that drives disease [194, 195].

A genotype distribution which gives rise to individuals with deleterious traits can be maintained by natural selection if the risk of producing costly extreme phenotypes is paid for by positive benefits of moderate alleles. When the detrimental phenotype is at the high end of the distribution and its onset is sudden, this is known as cliff edge selection [196]. The requirements for cliff edge selection are that the fitness function is asymmetric and the optimal distribution of genotypes in the population includes a tail of affected individuals, falling off the metaphorical ‘cliff’ [197]. This dynamic has been used to explain the persistence of deleterious heritable traits such as cephalopelvic disproportion [198], allergy [193], and schizophrenia [191, 199]. While the direction and strength of selection on HG risk alleles is not well known, genomic regions associated with heritable HG risk show elevated evolutionary conservation [50], consistent with a stabilizing selection regime such as cliff-edge selection. Together, available evidence strongly suggests that pregnancy sickness has an asymmetric fitness function, with increasing fitness benefits at intermediate levels and a turning point at which hyperemesis gravidarum risk greatly reduces fitness (Fig. 5).

### Evolutionary mismatch

Environmental mismatch may also contribute to hyperemesis gravidarum. Several major diseases, such as allergy, autoimmunity, obesity, and diseases of aging, either arose or worsened under the ‘transition to modernity,’ or the a shift toward sterile environments following industrialization [200–202]. Costly immunity defenses, greedy metabolic systems, and antagonistic pleiotropic adaptations for early life fecundity at the expense of longevity were acceptable trade-offs in a past environment characterized by high communicable disease risk, food and resource scarcity, and high extrinsic mortality. In a modern environment lacking all three of these properties, the same traits contribute to pathology. This phenomenon is known as mismatch [203].

**Figure 6:**
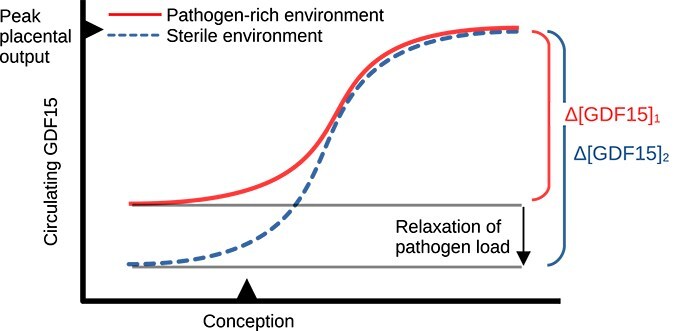
Mismatch model for increased hyperemesis gravidarum risk. Severity of NVP is related to the change between pre-pregnancy and post-pregnancy levels of circulating GDF15. In individuals with high parasitic load (solid curve), pre-pregnancy circulating GDF15 is more likely to be elevated above the levels in non-infected individuals in sterile living environments (dashed curve). The magnitude of the pregnancy GDF15 spike (Δ[GDF15]) is increased in the non-parasitized group.

Lower baseline GDF15 activation due to reduced parasite and toxin exposure may increase NVP severity and hyperemesis gravidarum risk. Women with elevated GDF15 levels before pregnancy—regardless of cause, whether toxin-induced, infection-induced, or from chronic conditions—show less severe nausea during pregnancy [22]. GDF15 is elevated by exposure to toxins including those in tobacco smoke [204]: smokers have increased serum GDF15 levels over non-smokers, estimated in one study to amount to a 36% increase [205], and maternal smoking is associated with a significantly reduced risk of hyperemesis gravidarum relative to non-smokers (OR = 0.40; 95% CI: 0.24–0.56) [206]. Serum GDF15 is also elevated by parasitic infection [47, 207]. While quantitative data from human parasitic infections are currently lacking, mouse models of *Toxoplasma gondii* infection show between 4.7-fold [207] and 7.5-fold [47] elevations of serum GDF15 one week after infection, and that levels remain above twice baseline for at least 2 weeks. Women with beta thalassemia, a condition associated with persistently high circulating GDF15 levels, also show a more than 90% reduction in incidence of NVP [22, 208]. Throughout much of human and catarrhine evolution, parasite load and environmental toxin exposure was likely higher than current levels. In these environmental conditions when placental GDF15 production first evolved, hyperemesis gravidarum risk would have been relatively low [209]. The sensitivity of the GDF15-GFRAL/RET signaling axis may have been evolutionarily calibrated to a more modest pregnancy fold-change than occurs in modern living conditions, leading to mismatch (Fig. 6).

Cross-cultural comparison is a route to testing the mismatch hypothesis. The hypothesis predicts that (i) populations with higher parasite load should have lower incidence of NVP, that (ii) populations in which NVP is reportedly completely absent should have significantly higher pathogen burdens, or greater exposure to toxins such as tobacco, than those with regular NVP, and conversely that (iii) populations with high prevalence of hyperemesis gravidarum should have low prevalence of environmental pathogens. Eight world populations, mostly small hunter-gatherer or subsistence farming communities, have been documented not to experience NVP, while at least 20 small populations in comparable conditions are documented to experience NVP and recognize it culturally [11]. Those reported to lack NVP are not geographically clustered or closely related [10]. The mere existence of pregnancy sickness is therefore not a disease of modernity, but the influence of lifestyle factors on its severity needs further investigation. The eight cultures without NVP have in common high levels of maize in the diet and low levels of meat consumption [10]. Attempts to correlate NVP prevalence with diet across cultures revealed patterns that are robust within world regions, but not between, suggesting that important confounding factors have yet to be identified [133]; GDF15 research suggests that environmental pathogen prevalence and smoking rates are two yet unstudied confounding factors needing consideration. Epidemiological data on pregnancy sickness in South America, South Asia, and Africa are extremely limited. More research in these regions, and in the developing world, is necessary to examine inter-population differences and to avoid projecting patterns from Western, industrialized, and wealthy nations as human universals.

### Parent-offspring conflict

Conflict is a recurrent theme in reproduction, arising because maternal and fetal (or paternal) optima for resource allocation are not entirely aligned [19, 210]. Several theories link NVP to such conflict. ‘Current conflict’ models propose that maternal nausea is greatest when embryonic quality lies on the border of rejection, and is caused by pro-gestational embryonic signaling being so strong as to compromise the health of the mother, resulting in nausea [16]. The current conflict model for NVP is not mechanistically supported and predicts an association between low embryonic viability and nausea which is not empirically observed [18].

‘Historical conflict’ models instead view NVP as the long-term outcome of escalating fetal strategies to ensure acceptance or secure investment at the mother’s expense. Historical conflict is exemplified by Crespi’s [17] proposal that fetal GDF15 mobilizes maternal glucose stores. Historical conflict predicts co-evolutionary escalation between the fetus and mother, where maternal sensitivity declines and fetal signaling strength increases [163]. This leads to precarious situations where, if maternal counter-adaptations are taken away, an extreme phenotype can result; this dynamic contributes to disorders such as gestational diabetes and pre-eclampsia [19]. GDF15 is a good candidate for an escalated signal, as it appears to have undergone a > 100× increase in expression in the catarrhine lineage (Fig. 4); within this framework hyperemesis gravidarum would be analogous to other diseases of conflict. Species with high levels of placental GDF15 production are predicted to also have acquired maternal adaptations leading to reduced sensitivity to its effects—a common pattern in conflict-driven evolutionary arms races.

Conflict exacerbates traits under cliff-edge selection (Fig. 5) whenever the pathological extreme state affects the sexes, or mother and fetus, disproportionately [211]. In the classic model of obstructed birth: a smaller pelvis gives ambulatory benefits to both sexes, but females disproportionately bear its costs in childbirth; modeling suggests that this sexual conflict increases the evolutionarily optimal level of obstruction risk beyond that if both sexes were equally affected [198]. Similarly, alleles conferring sensitivity to GDF15 are beneficial to both sexes in the response to infection, but the cost of high GDF15 sensitivity in pregnancy is disproportionately paid by females. A greater paternal optimum for GDF15 signaling strength should increase the acceptable proportion of genotypes falling off of the hyperemesis ‘cliff’ (Fig. 5). This pattern restricts natural selection from decreasing the proportion of individuals at risk.

Prophylactic toxin-avoidance theories assume cooperation rather than conflict: Profet’s [29] book is subtitled ‘using your body’s natural defenses to protect your baby-to-be’, and Flaxman and Sherman’s [10] expansion of the theory is subtitled ‘a mechanism for protecting mother and embryo’. Because teratogens harm fetuses more than mothers, mothers have more to lose from exercising excessive caution in the form of opportunities for high-value foods like meat; this could help explain why the fetus is the party that enforces avoidance, but is not a compelling conflict dynamic alone as it should be expected that interests are aligned in the vast majority of cases. Additionally, if GDF15’s primary effects are on other fetal cells as in the placental growth and development hypothesis, there is less opportunity for conflict than with fetal–maternal signaling. Overall, conflict plausibly contributes to selection maintaining NVP and escalated hyperemesis gravidarum risk in several of the non-prophylactic adaptive scenarios for placental GDF15, and indirectly to the prophylactic hypothesis through disproportionate costs of GDF15 sensitivity between the sexes. Historical conflict models for NVP are better supported than current conflict.

## RELEVANCE TO CLINICAL PRACTICE

The discovery of placental GDF15 as a proximate driver of NVP and hyperemesis gravidarum has opened new therapeutic possibilities. Proposed interventions include blocking GFRAL/RET, neutralizing circulating GDF15 with antibodies, or pre-dosing high-risk individuals with recombinant GDF15 to blunt the pregnancy surge.

Each approach has distinct implications for functional theories for NVP. With current technological means to sanitize food, it has been argued that any innate toxin avoidance function that NVP serves is vestigial and obsolete [14, 30]. Indeed, if behavioral prophylaxis is the sole function of NVP and is obsolete in modern humans due to sanitation and dietary safety, then any one of the proposed approaches should be clinically safe. However, if instead GDF15 has local functions at the fetal-maternal interface, as suggested by the anti-rejection and placental growth hypotheses, then neutralization of GDF15 directly could carry a greater risk of side effects than a more precise targeting of brainstem GFRAL.

Pre-dosing with GDF15 [22], is currently the only proposed therapy compatible with all major evolutionary hypotheses, as it would maintain protective local signals while also reducing the fold-change that triggers nausea. This mismatch-mitigation approach is conceptually analogous to worm therapy for autoimmune disease [212, 213]. The major practical limitation is that pre-dosing must be done before a patient becomes pregnant, and so would primarily benefit women with prior hyperemesis gravidarum or high hereditary risk. Which interventions prove appropriate will ultimately be determined in the clinic, rather than the evolutionist’s armchair.

The possibility that NVP has adaptive value at low levels should not dissuade interventions to improve human health. By analogy, cancer-risk-associated *BRCA* gene variants may have spread via natural selection [214], but preventative treatment to prevent the risk of serious pathology in individuals carrying these variants is entirely justified. With molecular and evolutionary understanding converging, prospects for transformative treatment of hyperemesis gravidarum, once dismissed, appears bright.

## CONCLUSIONS AND FUTURE DIRECTIONS

Explaining pregnancy sickness requires integrating proximate and ultimate causes. Recent advances have established that its proximate origins stem from placental production of the hormone GDF15, which activates a conserved neuroendocrine pathway regulating nausea and vomiting. Research into this pathway is revealing a surprisingly sophisticated system for toxin avoidance in non-reproductive contexts. Pregnancy sickness is thus not a wholly novel behavioral adaptation, but the acute activation of a pre-existing endocrine circuit by placental hormones. Comparative transcriptomic evidence suggests that high levels of placental GDF15 production evolved in Catarrhini, the apes and Old World monkeys, and is not restricted to humans. Claims that pregnancy sickness represents a distinct behavioral adaptation must clear a higher evidentiary bar, and narratives linking it to the peculiarities of human cultural and biological evolution require revision.

The popular prophylactic hypothesis was ahead of its time in recognizing the functional necessity of toxin avoidance, and that organisms have innate behaviors and physiological mechanisms to do so [14, 126]. However, as these mechanisms are more ancient, the original hypothesis appears to incorrectly identify the derived trait and so misattribute the main evolutionary driver of pregnancy sickness. All placental mammals would benefit from teratogen avoidance during fetal organogenesis, yet only Catarrhini show a dramatic upregulation of placental GDF15. There is no clear evidence of unique dietary toxins or embryonic vulnerability in Catarrhini, but what sets this lineage apart instead is a radical divergence in placental biology, specifically in the syncytiotrophoblast, the major cellular source of GDF15. Unless clear examples of convergent evolution of pregnancy-specific avoidance behaviors in other lineages are found, or sufficient differences separating pregnancy sickness behavior as a distinct trait from generalized sickness behavior (the ‘two trait model’ of Fig. 2), prophylaxis alone is likely insufficient to explain its origins.

Events in placental evolution may better explain the selection for placental GDF15 production, whether promoting placental growth, modulating maternal tolerance, or adjusting maternal metabolism. Adjudication between these evolutionary theories will require further knowledge of GDF15’s proximate effects. Whether placental GDF15 can also exert local, non-brainstem-mediated effects at the fetal-maternal interface, and whether these effects significantly influence fetal survival or birthweight, remain crucial knowledge gaps. Other hormones such as leptin, FGF21, and insulin were once thought to have narrowly-defined receptors and activities, and later shown to act across multiple tissues and contexts [215–217]. GDF15 may yet surprise us. If non-canonical effects are rigorously demonstrated, it is possible that nausea and vomiting are byproducts or secondary functions, rather than primary functions, of placental GDF15.

Trade-offs and cliff-edge selection are plausible explanations for the evolutionary persistence of pathological pregnancy sickness or hyperemesis gravidarum, possibly exacerbated by reduced exposure in modern environments to pathogens and other GDF15 triggers. Epidemiological evidence testing the mismatch model of NVP is still lacking, and is an important area of future investigation.

Beyond its public health importance, pregnancy sickness is a powerful model for studying the evolution of heritable neuroendocrine traits and the interplay between physiological and behavioral host defenses. More than a curiosity of human evolution, pregnancy sickness offers unique insight into the genotype–phenotype map and the origins of complex adaptive behaviors.

## Supplementary Material

Supplementary_materials_eoaf025_Tables

Supplementary_Tables_eoaf025

Supplementary_Text_eoaf025

## Data Availability

No new data were generated for this study. Accession numbers of gene expression data used for plotting are available as Supplementary data.
