# Supplementary material for: Proximate and ultimate causes of pregnancy sickness
Source: Evol Med Public Health. 2025 Sep 18;13(1):307–30. doi: 10.1093/emph/eoaf025 (PMC12542990; doi:10.1093/emph/eoaf025)
Supplement: Supplementary_Text_eoaf025 [file supplementary_text_eoaf025.docx]

**Supplementary Text to:**

"Proximate and Ultimate Causes of Pregnancy Sickness."

Stadtmauer, 2025, *Evolution, Medicine, and Public Health*

**Methods for Whole Placenta RNA-sequencing Plotting (Figure 3)**

Bulk RNA sequencing records were retrieved from the NCBI Gene Expression Omnibus following a search for all entries from mammalian placentas (trophoblast/fetal cells only, not decidua). Samples from commonly-studied species which were redundant with others (e.g. human, mouse) were excluded. Reads were quantified using the pseudoaligment tool kallisto (v0.51.1) against the species' respective genome in the Ensembl Database Version 113, or if not included in Ensembl, its NCBI genome or Ensembl Beta. Samples retrieved and genomes used are reported in Supplementary Table 1. Gene abundances (transcripts per million; TPM) were subset to only those genes present in annotations of all species compared, leaving a total of 5096 genes. TPM values were re-normalized to 1 million for each sample and square-root transformed for plotting.
